# Supplementary material for: Mechanisms governing the pioneering and redistribution capabilities of the non-classical pioneer PU.1
Source: Nat Commun. 2020 Jan 21;11:402. doi: 10.1038/s41467-019-13960-2 (PMC6972792; doi:10.1038/s41467-019-13960-2)
Supplement: Supplementary file 7 — Source data [file 41467_2019_13960_MOESM7_ESM.zip › Source_Data/FigureS2/FigureS2B_MotifScanOutput/KmeansCluster6/homerResults.html]

/misc/data/analysis/project\_PU1/CTV1/motifs/basic/PU1\_Kmeans14.6/final - Homer de novo Motif Results


# Homer *de novo* Motif Results (/misc/data/analysis/project\_PU1/CTV1/motifs/basic/PU1\_Kmeans14.6/final)

Known Motif Enrichment Results  
Gene Ontology Enrichment Results  
If Homer is having trouble matching a motif to a known motif, try copy/pasting the matrix file into
STAMP  
More information on motif finding results: HOMER
| Description of Results
| Tips
  
Total target sequences = 3293  
Total background sequences = 43004  
\* - possible false positive  

|  |  |  |  |  |  |  |  |  |
| --- | --- | --- | --- | --- | --- | --- | --- | --- |
| Rank | Motif | P-value | log P-pvalue | % of Targets | % of Background | STD(Bg STD) | Best Match/Details | Motif File |
| 1 | A G T C C T G A A T G C C G A T A C G T A G T C A G T C A G C T A G T C G C A T G C A T G A C T | 1e-1720 | -3.961e+03 | 87.52% | 14.32% | 36.0bp (84.0bp) | PB0058.1\_Sfpi1\_1/Jaspar(0.963) More Information \| Similar Motifs Found | motif file (matrix) |
| 2 | C G T A A T C G A C G T A C G T A G T C A G C T A G T C | 1e-346 | -7.989e+02 | 47.34% | 16.06% | 54.1bp (76.9bp) | PB0099.1\_Zfp691\_1/Jaspar(0.750) More Information \| Similar Motifs Found | motif file (matrix) |
| 3 | A T G C G A C T A G C T A G T C G A T C G C A T A T G C G A C T A G C T T A G C G A T C G A C T A G T C | 1e-310 | -7.148e+02 | 50.14% | 19.39% | 47.4bp (88.7bp) | ELF3(ETS)/PDAC-ELF3-ChIP-Seq(GSE64557)/Homer(0.694) More Information \| Similar Motifs Found | motif file (matrix) |
| 4 | C T G A A T G C C G A T C A G T A G T C C G A T A T C G G A T C G C A T G C A T G A C T G A C T | 1e-223 | -5.156e+02 | 20.10% | 4.01% | 42.3bp (76.6bp) | SpiB(ETS)/OCILY3-SPIB-ChIP-Seq(GSE56857)/Homer(0.784) More Information \| Similar Motifs Found | motif file (matrix) |
| 5 | C T A G A T G C C G A T A C G T G T A C A G T C G A T C G A C T | 1e-131 | -3.026e+02 | 24.81% | 9.42% | 50.5bp (78.8bp) | GCR2(MacIsaac)/Yeast(0.778) More Information \| Similar Motifs Found | motif file (matrix) |
| 6 | C G A T C A T G C G T A C A T G A C T G G C T A G T C A T G A C G C A T C A T G G C T A | 1e-126 | -2.910e+02 | 15.97% | 4.38% | 58.1bp (73.1bp) | PU.1(ETS)/ThioMac-PU.1-ChIP-Seq(GSE21512)/Homer(0.742) More Information \| Similar Motifs Found | motif file (matrix) |
| 7 | T A G C C G T A C T A G G C A T C A G T G A T C A G C T A G T C T C G A A G C T C T A G C T G A C A T G T C G A | 1e-96 | -2.217e+02 | 6.35% | 0.79% | 41.9bp (76.4bp) | ZBTB33(Zf)/GM12878-ZBTB33-ChIP-Seq(GSE32465)/Homer(0.722) More Information \| Similar Motifs Found | motif file (matrix) |
| 8 | T C A G C T G A G A C T C A G T T C A G G A C T T C G A C G T A T C A G G C A T A G C T G A C T A G T C G A T C | 1e-78 | -1.818e+02 | 4.34% | 0.39% | 72.3bp (71.8bp) | tll/dmmpmm(Papatsenko)/fly(0.659) More Information \| Similar Motifs Found | motif file (matrix) |
| 9 | A C G T A G T C G T C A A G C T C T A G T G C A A C T G C T G A C G T A A T G C T G C A C T A G G T A C C G T A | 1e-75 | -1.737e+02 | 3.95% | 0.32% | 52.6bp (58.8bp) | Ot\_0263(RRM)/Ostreococcus\_tauri-RNCMPT00263-PBM/HughesRNA(0.703) More Information \| Similar Motifs Found | motif file (matrix) |
| 10 | T C A G C G A T T C A G T G C A A G C T C G T A A T C G C G A T T C A G C T G A A T C G A C G T | 1e-70 | -1.613e+02 | 4.62% | 0.57% | 55.5bp (71.9bp) | FZF1/MA0298.1/Jaspar(0.676) More Information \| Similar Motifs Found | motif file (matrix) |
| 11 | G T C A A G T C G T C A G T A C T C G A G T A C A C G T A G C T A C G T G A C T T C G A C G T A G C T A G T A C | 1e-66 | -1.531e+02 | 3.16% | 0.21% | 25.7bp (68.1bp) | AT1G69570(C2C2dof)/col-AT1G69570-DAP-Seq(GSE60143)/Homer(0.656) More Information \| Similar Motifs Found | motif file (matrix) |
| 12 | T C A G C T A G A T C G C T A G C T G A C T A G A C T G G T C A C G T A T A C G T G A C | 1e-66 | -1.525e+02 | 13.42% | 5.12% | 47.6bp (76.4bp) | GCR2(MacIsaac)/Yeast(0.803) More Information \| Similar Motifs Found | motif file (matrix) |
| 13 | C T A G A G T C C G A T A C G T G T A C G T A C G A T C A T G C A C G T A C G T A G C T A C T G A G T C A G T C | 1e-63 | -1.454e+02 | 2.55% | 0.11% | 50.2bp (58.3bp) | SpiB(ETS)/OCILY3-SPIB-ChIP-Seq(GSE56857)/Homer(0.665) More Information \| Similar Motifs Found | motif file (matrix) |
| 14 | A T G C A G T C A C G T A G C T A G T C G A T C C T G A G A T C A G T C C G T A A C G T C T A G | 1e-62 | -1.441e+02 | 6.26% | 1.33% | 65.2bp (68.5bp) | Tv\_0259(RRM)/Trichomonas\_vaginalis-RNCMPT00259-PBM/HughesRNA(0.662) More Information \| Similar Motifs Found | motif file (matrix) |
| 15 | C G T A A G C T A C G T C T A G C T A G T C G A G C A T T A G C T C G A A G C T C T A G A C T G T C A G C A T G | 1e-62 | -1.439e+02 | 3.77% | 0.40% | 73.9bp (67.4bp) | Atf4(bZIP)/MEF-Atf4-ChIP-Seq(GSE35681)/Homer(0.690) More Information \| Similar Motifs Found | motif file (matrix) |
| 16 | G C A T C T A G A C G T A C G T A C G T A G T C A T G C A C G T A T C G | 1e-56 | -1.306e+02 | 11.51% | 4.37% | 51.4bp (70.1bp) | EWS:ERG-fusion(ETS)/CADO\_ES1-EWS:ERG-ChIP-Seq(SRA014231)/Homer(0.804) More Information \| Similar Motifs Found | motif file (matrix) |
| 17 | A T G C A C G T A C G T A T G C A G T C A C G T A T C G A C G T | 1e-56 | -1.293e+02 | 10.32% | 3.67% | 58.9bp (74.6bp) | Ets1-distal(ETS)/CD4+-PolII-ChIP-Seq(Barski\_et\_al.)/Homer(0.903) More Information \| Similar Motifs Found | motif file (matrix) |
| 18 | T G C A A C G T A G C T G C A T T A G C G A T C T A G C A G C T A G C T | 1e-55 | -1.279e+02 | 11.66% | 4.52% | 54.0bp (77.9bp) | RNP4F(RRM)/Drosophila\_melanogaster-RNCMPT00060-PBM/HughesRNA(0.675) More Information \| Similar Motifs Found | motif file (matrix) |
| 19 | A T C G G T C A C T G A T A C G C G T A C T G A A C T G T C G A C G T A C T A G T A C G C G T A | 1e-53 | -1.227e+02 | 9.29% | 3.19% | 53.0bp (82.3bp) | TRA2(RRM)/Drosophila\_melanogaster-RNCMPT00078-PBM/HughesRNA(0.700) More Information \| Similar Motifs Found | motif file (matrix) |
| 20 | C G T A C T A G C T A G C T A G C T A G C T A G C G T A G T C A T C G A C G A T G T A C G A T C T C G A A G T C | 1e-51 | -1.176e+02 | 1.88% | 0.06% | 64.6bp (40.7bp) | dl(var.2)/MA0023.1/Jaspar(0.733) More Information \| Similar Motifs Found | motif file (matrix) |
| 21 | T G A C C T G A C G T A C T G A T A G C C G T A C T A G C T A G A C T G C T G A C G T A A T C G G C A T | 1e-48 | -1.128e+02 | 3.28% | 0.42% | 41.1bp (77.8bp) | ELF5(ETS)/T47D-ELF5-ChIP-Seq(GSE30407)/Homer(0.765) More Information \| Similar Motifs Found | motif file (matrix) |
| 22 | C T G A T C G A A C T G C T G A C G A T A G T C A C G T A C T G C T A G A G C T A C T G A C T G A C G T A C G T | 1e-44 | -1.033e+02 | 2.92% | 0.35% | 40.5bp (73.2bp) | JGL(C2H2)/col-JGL-DAP-Seq(GSE60143)/Homer(0.673) More Information \| Similar Motifs Found | motif file (matrix) |
| 23 | A G T C A C G T A T G C A C G T A G T C A G T C A G T C A C G T A G T C A G T C C G A T C A T G G A T C | 1e-43 | -1.004e+02 | 4.37% | 0.93% | 43.7bp (88.3bp) | SeqBias: GA-repeat(0.723) More Information \| Similar Motifs Found | motif file (matrix) |
| 24 | A C G T A T C G G T A C A T C G C T G A C G T A A T G C G A C T T A G C | 1e-43 | -9.984e+01 | 3.58% | 0.61% | 56.5bp (80.5bp) | lin-14/MA0261.1/Jaspar(0.707) More Information \| Similar Motifs Found | motif file (matrix) |
| 25 | A C G T A G T C C G T A A T G C A C G T T A G C C G T A A G T C A C G T C G T A | 1e-43 | -9.917e+01 | 2.52% | 0.26% | 51.3bp (89.1bp) | z/dmmpmm(Down)/fly(0.740) More Information \| Similar Motifs Found | motif file (matrix) |
| 26 | G A T C C T G A C T G A A G T C A G T C C G T A A T G C C G T A | 1e-42 | -9.831e+01 | 19.68% | 11.06% | 53.0bp (67.4bp) | RUNX2(Runt)/PCa-RUNX2-ChIP-Seq(GSE33889)/Homer(0.947) More Information \| Similar Motifs Found | motif file (matrix) |
| 27 | C G T A C T G A A G T C G T C A A C T G A T G C G T C A | 1e-42 | -9.728e+01 | 25.90% | 16.11% | 56.8bp (71.4bp) | ACE2/ACE2\_YPD/2-SWI5(Harbison)/Yeast(0.790) More Information \| Similar Motifs Found | motif file (matrix) |
| 28 | G T A C A C G T T A C G C T A G C G A T C T A G C T A G C T A G G T C A C T A G C T A G A C G T C T A G | 1e-41 | -9.547e+01 | 2.00% | 0.14% | 81.8bp (71.6bp) | AtMYB84(MYB)/Arabidopsis thaliana/AthaMap(0.696) More Information \| Similar Motifs Found | motif file (matrix) |
| 29 | A G C T C T A G A G T C A G T C C G T A A G C T T C A G C G A T C T A G C G T A | 1e-41 | -9.467e+01 | 10.90% | 4.79% | 52.8bp (65.7bp) | INO4/MA0322.1/Jaspar(0.804) More Information \| Similar Motifs Found | motif file (matrix) |
| 30 | A C G T C A T G A T C G C G A T A C G T A T C G A C G T A C G T C G A T C T G A C G T A C G T A | 1e-40 | -9.412e+01 | 2.34% | 0.23% | 38.6bp (65.6bp) | HCM1/MA0317.1/Jaspar(0.670) More Information \| Similar Motifs Found | motif file (matrix) |
| 31 | C G A T A C G T A G C T G T A C G T A C A G C T A G T C A C G T C G A T A C G T A G T C G C T A | 1e-40 | -9.303e+01 | 3.04% | 0.46% | 46.0bp (66.2bp) | PI/MA0559.1/Jaspar(0.777) More Information \| Similar Motifs Found | motif file (matrix) |
| 32 | A T C G A G T C G A C T C T G A G T A C C G T A G A T C C G T A T G A C C A G T | 1e-36 | -8.513e+01 | 2.70% | 0.40% | 32.5bp (73.7bp) | Rbm38(RRM)/Danio\_rerio-RNCMPT00283-PBM/HughesRNA(0.727) More Information \| Similar Motifs Found | motif file (matrix) |
| 33 | C A T G A C T G C T G A C T G A A T C G C G T A G A C T C T A G A G C T T A C G A G T C | 1e-35 | -8.276e+01 | 2.55% | 0.36% | 43.0bp (78.8bp) | twi/dmmpmm(Pollard)/fly(0.691) More Information \| Similar Motifs Found | motif file (matrix) |
| 34 | C G T A C T A G C T A G A G T C G T A C A C G T A G T C C T G A C T A G A C T G G T C A | 1e-31 | -7.258e+01 | 3.04% | 0.62% | 76.3bp (75.9bp) | ZFX(Zf)/mES-Zfx-ChIP-Seq(GSE11431)/Homer(0.723) More Information \| Similar Motifs Found | motif file (matrix) |
| 35 | A G T C T G A C T C G A A T G C T C G A G C A T T C A G G T C A | 1e-31 | -7.225e+01 | 12.09% | 6.27% | 56.2bp (69.1bp) | TFE3/MA0831.1/Jaspar(0.749) More Information \| Similar Motifs Found | motif file (matrix) |
| 36 | A C G T A G T C A C G T A C T G A G T C A G T C C G T A A C G T A T C G C T A G | 1e-31 | -7.221e+01 | 2.46% | 0.40% | 55.3bp (78.3bp) | RIM101/Literature(Harbison)/Yeast(0.688) More Information \| Similar Motifs Found | motif file (matrix) |
| 37 | A G C T A C G T C G A T G A C T G C T A C T G A C G T A C T G A A G T C A G T C C G T A C G A T A T G C | 1e-30 | -7.004e+01 | 2.52% | 0.44% | 37.1bp (69.4bp) | HuR(RRM)/Homo\_sapiens-RNCMPT00136-PBM/HughesRNA(0.735) More Information \| Similar Motifs Found | motif file (matrix) |
| 38 | G A T C G T A C T A C G G A T C A T G C C G T A A G C T C T A G | 1e-29 | -6.778e+01 | 9.99% | 4.91% | 63.7bp (68.6bp) | YY2/MA0748.1/Jaspar(0.775) More Information \| Similar Motifs Found | motif file (matrix) |
| 39 | A C T G C G T A C T A G A C T G A G C T A C T G C G T A A G C T A C G T A C T G | 1e-27 | -6.397e+01 | 2.10% | 0.32% | 77.2bp (72.4bp) | Dux/MA0611.1/Jaspar(0.716) More Information \| Similar Motifs Found | motif file (matrix) |
| 40 | C G T A A C T G A T C G C T G A C T A G A C T G G T C A G T C A A C T G A C T G C G T A | 1e-26 | -6.156e+01 | 3.13% | 0.78% | 45.9bp (91.3bp) | SRSF9(RRM)/Homo\_sapiens-RNCMPT00067-PBM/HughesRNA(0.763) More Information \| Similar Motifs Found | motif file (matrix) |
| 41 | A G C T T G A C C T G A G A C T A C T G C T G A A C T G C G A T | 1e-25 | -5.986e+01 | 5.04% | 1.85% | 49.6bp (70.6bp) | Tv\_0259(RRM)/Trichomonas\_vaginalis-RNCMPT00259-PBM/HughesRNA(0.759) More Information \| Similar Motifs Found | motif file (matrix) |
| 42 | A C G T A T G C A C T G A C G T A C T G A C T G C T A G C G T A | 1e-25 | -5.952e+01 | 6.89% | 3.02% | 65.6bp (70.2bp) | Su(H)/dmmpmm(Papatsenko)/fly(0.790) More Information \| Similar Motifs Found | motif file (matrix) |
| 43 | C G A T A G T C A G C T T G A C C A T G A G T C A T G C C G A T C T A G A T G C A G C T | 1e-25 | -5.883e+01 | 3.70% | 1.11% | 45.8bp (76.5bp) | BPC1(BBRBPC)/colamp-BPC1-DAP-Seq(GSE60143)/Homer(0.642) More Information \| Similar Motifs Found | motif file (matrix) |
| 44 | T C A G G T A C G A T C T C G A A G C T T C G A G C A T C T A G | 1e-24 | -5.716e+01 | 8.75% | 4.36% | 51.6bp (63.4bp) | Twist2/MA0633.1/Jaspar(0.869) More Information \| Similar Motifs Found | motif file (matrix) |
| 45 | C G T A A C G T A T G C A C G T A C T G C T A G A C G T | 1e-23 | -5.359e+01 | 21.17% | 14.39% | 52.4bp (67.8bp) | CG14718(RRM)/Drosophila\_melanogaster-RNCMPT00006-PBM/HughesRNA(0.802) More Information \| Similar Motifs Found | motif file (matrix) |
| 46 | C G A T C G T A C G T A A G T C T A G C C T G A A T G C A G T C | 1e-23 | -5.358e+01 | 13.79% | 8.31% | 53.2bp (66.6bp) | Gam1/MA0034.1/Jaspar(0.829) More Information \| Similar Motifs Found | motif file (matrix) |
| 47 | C G T A G T C A A C T G A G C T C T A G A C G T A C T G | 1e-22 | -5.282e+01 | 11.87% | 6.84% | 48.6bp (70.4bp) | RBM24(RRM)/Homo\_sapiens-RNCMPT00184-PBM/HughesRNA(0.836) More Information \| Similar Motifs Found | motif file (matrix) |
| 48 | C G T A G C T A A T C G C G T A C T A G T A C G G T C A C G T A A T C G C T G A C G T A C T A G C G T A C G T A | 1e-22 | -5.163e+01 | 2.40% | 0.55% | 49.5bp (95.6bp) | TRA2(RRM)/Drosophila\_melanogaster-RNCMPT00078-PBM/HughesRNA(0.708) More Information \| Similar Motifs Found | motif file (matrix) |
| 49 | G A T C G T C A A C G T C A T G T A C G A G C T C T G A C G T A C T A G C G T A G A T C T C A G G A C T A C T G | 1e-22 | -5.145e+01 | 0.85% | 0.03% | 45.1bp (64.3bp) | Tv\_0259(RRM)/Trichomonas\_vaginalis-RNCMPT00259-PBM/HughesRNA(0.639) More Information \| Similar Motifs Found | motif file (matrix) |
| 50 | A C G T A C G T A G C T A C T G G T A C A G T C A C G T C G A T | 1e-19 | -4.453e+01 | 9.66% | 5.49% | 59.4bp (68.2bp) | tll/MA0459.1/Jaspar(0.772) More Information \| Similar Motifs Found | motif file (matrix) |
| 51 | A G C T C T A G A T C G A G T C C G A T C A T G C A T G C T A G C A T G C G T A C T A G C T A G A G T C G A T C | 1e-18 | -4.320e+01 | 1.82% | 0.38% | 70.5bp (76.4bp) | ZNF416(Zf)/HEK293-ZNF416.GFP-ChIP-Seq(GSE58341)/Homer(0.646) More Information \| Similar Motifs Found | motif file (matrix) |
| 52 | A C G T A C T G C G T A A C G T A G T C C G A T A G T C A C G T A C T G A G T C C G T A A G T C C G T A A G C T | 1e-18 | -4.252e+01 | 0.55% | 0.01% | 30.8bp (46.8bp) | PB0104.1\_Zscan4\_1/Jaspar(0.646) More Information \| Similar Motifs Found | motif file (matrix) |
| 53 | A G T C A C G T A G T C A G T C A C G T A T G C A T G C A C G T A G T C | 1e-17 | -4.045e+01 | 5.19% | 2.40% | 45.9bp (83.7bp) | SRSF1(RRM)/Homo\_sapiens-RNCMPT00163-PBM/HughesRNA(0.841) More Information \| Similar Motifs Found | motif file (matrix) |
| 54 | A C T G C T A G A C T G C T A G A G T C A T G C A C G T T A C G C T A G A C G T | 1e-16 | -3.852e+01 | 1.79% | 0.42% | 73.7bp (80.5bp) | ZNF711(Zf)/SHSY5Y-ZNF711-ChIP-Seq(GSE20673)/Homer(0.722) More Information \| Similar Motifs Found | motif file (matrix) |
| 55 | T C A G C T A G A G C T A C G T A C G T A G T C A G T C A G T C T G A C G T A C T C G A | 1e-16 | -3.773e+01 | 1.15% | 0.17% | 64.8bp (54.0bp) | Su(H)/MA0085.1/Jaspar(0.763) More Information \| Similar Motifs Found | motif file (matrix) |
| 56 | A T G C A T C G C A T G T C G A C T A G C T A G A G T C G A T C G C A T T A G C | 1e-15 | -3.474e+01 | 2.92% | 1.08% | 75.3bp (69.7bp) | ZFX(Zf)/mES-Zfx-ChIP-Seq(GSE11431)/Homer(0.726) More Information \| Similar Motifs Found | motif file (matrix) |
| 57 | C T G A A C G T G A C T C G T A A T C G G A C T T C G A G T A C C G T A T C A G A G T C A G T C | 1e-13 | -3.078e+01 | 0.67% | 0.05% | 38.6bp (38.7bp) | PB0096.1\_Zfp187\_1/Jaspar(0.716) More Information \| Similar Motifs Found | motif file (matrix) |
| 58 | A C T G C G T A A C G T A C G T A C T G A C T G C G T A A C G T | 1e-13 | -3.059e+01 | 1.61% | 0.43% | 76.6bp (74.0bp) | HAP2/Literature(Harbison)/Yeast(0.791) More Information \| Similar Motifs Found | motif file (matrix) |
| 59 | A C T G A C T G G T C A C T G A C T A G T A C G C G T A A C T G C G T A C T G A C T A G G T C A G T C A A C T G | 1e-13 | -3.031e+01 | 1.34% | 0.30% | 51.0bp (65.9bp) | Unknown4/Arabidopsis-Promoters/Homer(0.706) More Information \| Similar Motifs Found | motif file (matrix) |
| 60 | C G T A C G T A A C G T A G T C C G T A A C G T A C T G | 1e-12 | -2.956e+01 | 5.56% | 3.01% | 66.6bp (67.4bp) | HAT1/MA1024.1/Jaspar(0.899) More Information \| Similar Motifs Found | motif file (matrix) |
| 61 | C T A G A G T C C G T A C T G A A T C G A G T C C G T A | 1e-12 | -2.888e+01 | 8.53% | 5.33% | 55.8bp (71.6bp) | MBNL1(Znf)/Homo\_sapiens-RNCMPT00038-PBM/HughesRNA(0.918) More Information \| Similar Motifs Found | motif file (matrix) |
